# Study on the Carcass Traits, Meat Quality, and Nutritional Attributes of Six Kinds of Jiangxi Local Breeds Cattle

**DOI:** 10.3390/ani14213053

**Published:** 2024-10-22

**Authors:** Yang Zhang, Yuting Wei, Guwei Lu, Youxiang Yang, Yuting Pan, Chuanpei Fu, Fazhan Tian, Qinghua Qiu, Xianghui Zhao, Yanjiao Li, Lingli Chen, Wenjun Wang, Kehui Ouyang

**Affiliations:** 1 Jiangxi Province Key Laboratory of Animal Nutrition/Animal Nutrition and Feed Safety Innovation Team, College of Animal Science and Technology, Jiangxi Agricultural University, Nanchang 330045, China; jiushixiaoyang77@sina.com (Y.Z.); weiyutingview@163.com (Y.W.); lgw0202020245@163.com (G.L.); yangyxjessie@163.com (Y.Y.); sa2716038184@163.com (Y.P.); fuchuanpei123@outlook.com (C.F.); 18070501275@163.com (F.T.); zhaoxh001@163.com (X.Z.); yanjiaoli221@jxau.edu.cn (Y.L.); 2 Key Lab for Agro-Product Processing and Quality Control of Nanchang City, College of Food Science and Engineering, Jiangxi Agricultural University, Nanchang 330045, China; chenlingli89@163.com

**Keywords:** Jiangxi breed cattle, carcass characteristic, meat quality, nutritional ingredients, muscle fiber characteristics

## Abstract

The purpose of this study is to explore the carcass traits, meat quality, and nutritional attributes of local yellow cattle (Guangfeng, Ji’an, and Jinjiang) and buffalo (Poyanghu, Xiajiang, and Xinfeng mountain) in Jiangxi Province, and compare the differences among different breeds. The results showed that the dressing percentage, net meat percentage, and meat–bone ratio of Jinjiang cattle were highest, and that Jinjiang cattle had the best meat production performance. Regarding meat quality, the pH_24h_ value of all breeds was at normal level, and no dark-cutting beef was produced. Poyanghu buffalo and Xinfeng mountain buffalo had higher redness (a*) values, and Guangfeng cattle had the best water retention and tenderness. The intermuscular fat of yellow cattle breeds was higher than that of buffalo breeds; Poyanghu buffalo had the highest crude protein content. The composition of amino acids and fatty acids was different among breeds, and the composition of Jiangxi breeds was healthy. The muscle fibers of buffalo breeds are generally smaller and denser than those of yellow cattle breeds. The *MyHC-Ⅰ*, *MyHC-Ⅱa*, and *MyHC-Ⅱx* were the main types of muscle fibers of Jiangxi local breeds, and the proportion varied with different breeds. This work elucidated the carcass characteristics, meat quality, and nutritional attributes of Jiangxi breed cattle to provide a theoretical basis for optimizing the development of beef cattle industry.

## Simple Summary

China’s vast territory and varied terrain formed a rich resource of cattle breeds, but was limited by agricultural production methods until the 1970s when it began to shift in the direction of meat. In the past ten years, China’s beef cattle breeding has entered a new period of combined breeding, and the development and utilization of local genetic resources have been emphasized and strengthened. In this study, six local cattle breeds distributed in Jiangxi Province were selected to compare and measure their carcass characteristics, meat quality, and nutritional attributes, aiming to understand the production characteristics of local cattle in Jiangxi Province, provide references for beef cattle breeding programs, make up the gaps in previous studies, and provide certain references and create certain conditions for the protection and development of local cattle breeds.

## 1. Introduction

Due to China’s huge population and agricultural needs, animal husbandry is of great significance in promoting economic development and occupies an important position in agriculture. The cattle were the most important of the four main types of livestock in the Old World as it provided the following: meat, milk, leather, and could pull wagons, plow fields, and do other work. Previously, cattle were mainly used as draft animals, and only older ones were slaughtered for their meat [1]. However, with the progress of science and technology, agriculture underwent a process of gradual mechanization and modernization, and the role of cattle has also changed from a labor tool to a main provider of meat and milk [2]. With the continuous improvement of living standards, the consumption of beef in daily meals is rising. After a long period of selection and breeding, the meat production performance (especially yield and quality) of modern beef cattle breeds is better than that of their ancestors. In addition, the gradual development of buffalo as a meat resource has also eased the pressure on demand. In recent decades, the focus of China’s beef industry has been to increase beef production; however, with the improvement of meat productivity, consumers have begun to focus on the quality of meat. Quality has gradually become an important factor in determining consumer purchasing power; production and quality are both important to China’s beef industry today [3].

**Figure 1:**
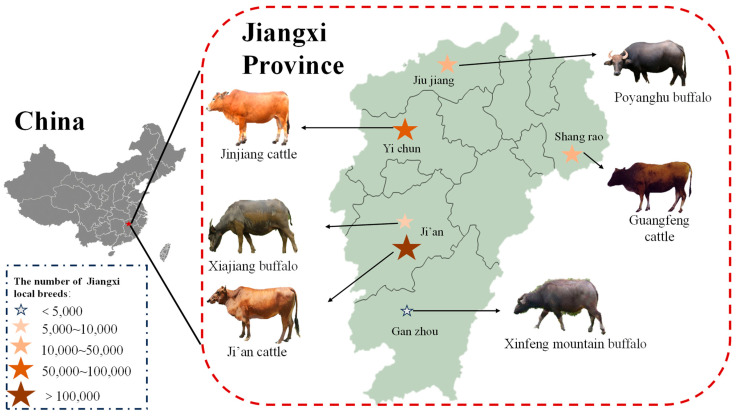
Distribution of local cattle breeds in Jiangxi.

The extant domesticated cattle can be divided into the following two major geographic groups: humpless taurine (*B. taurus*) and humped indicine (*B. indicus*), taurine cattle domesticated in the fertile Crescent and indicine cattle domesticated in the Indus Valley, respectively [4,5]. More than 1000 cattle breeds have been established worldwide through manual selection [6]. Of these varieties, 72 originated in China and are endemic to China. Among them, the buffalo is also known as the Asian animal and about 97% of the world’s buffalo live in the Asian region [7]. These Chinese breeds differ in their intrinsic characteristics and are an important genetic resource for cattle worldwide. Chinese native cattle have the advantages of parasite resistance, immune resistance, utilization of roughage-based diets, and tolerance to environmental challenges, and their broad geographical distribution rich genetic resources, animal adaptation, and environmental selection have shaped the genetic and epigenetic diversity of Chinese native cattle [8,9,10]. There are a series of performance differences in cattle growth and meat quality, and comparing these attributes across breeds can assess the production value of different cattle. It is well known that breed is an important factor affecting carcass characteristics, meat quality, and nutritional composition [11,12]. Jiangxi Province is located in the southeast of China, on the south bank of the middle and lower reaches of the Yangtze River. In terms of geographical environment, there are mountains and hills in the south and the Poyang Lake plain in the north. It has a mild climate and abundant meadow resources, which are suitable for herbivorous livestock breeding. The changeable geographical environment has bred six local cattle breeds. According to the origin, distribution, breed formation, and other factors, the local breeds in Jiangxi province can be divided into three kinds of cattle and three kinds of buffalo, namely Guangfeng cattle, Ji’an cattle, Jinjiang cattle, Poyanghu buffalo, Xiajiang buffalo, and Xinfeng mountain buffalo (Figure 1). Compared with other livestock, cattle grow at a slower rate and have lower economic benefits, so local people usually adopt the form of grazing. Grazing usually results in higher amino acids and flavor nucleotides in the meat of livestock and poultry, with better nutrition and flavor [13,14]. In addition, the number of cattle breeds in these places is gradually decreasing, facing great conservation pressure [15].

The beef cattle industry is an emerging industry that has developed rapidly in Jiangxi Province in recent years. Due to the constant change in consumption demand, beef cattle production is in short supply, which exposes the lack of excellent breeding support for Jiangxi’s beef cattle industry. Therefore, a comprehensive understanding of the carcass characteristics and meat quality of local cattle breeds is very important for the development of local beef cattle industry in Jiangxi. However, previous studies on Jiangxi cattle mainly focused on genetic breeding techniques and disease prevention and paid little attention to performance, meat quality, and nutritional composition [16,17]. As more attention has been paid to the related traits of meat breeds (such as Simmental and Angus) and crossbred cattle, less research has been performed on breeds in southeast China. This study selected six local cattle breeds distributed in Jiangxi Province and compared and measured their carcass characteristics, meat quality, and nutritional attributes. This study was designed to understand the production characteristics of local cattle in Jiangxi Province, provide references for beef cattle breeding programs, make up the gaps in previous studies, and provide certain references and create certain conditions for the protection and development of local cattle breeds.

## 2. Materials and Methods

The different local cattle breeds in Jiangxi (Guangfeng cattle, Ji’an cattle, Jinjiang cattle, Poyanghu buffalo, Xiajiang buffalo, and Xinfeng mountain buffalo) for grazing (*n* = 10, ♂:♀ = 5:5), aged 18–24 months, were transported to the commercial abattoir in advance for 7 d to alleviate transport stress. The Xinfeng mountain buffalo and Poyanghu buffalo in this study were identified and selected by the Jiangxi Cattle and Sheep System Expert Group, and the other varieties were from provincial or provincial temporary breeding farms. The animals used were weaned in June; grazed with their mothers; grazed for 10 h in spring, summer, and autumn; and grazed for 5 h and were supplemented with straw in winter. All animals were slaughtered following standard commercial procedures (NY 467-2001, Ministry of Agriculture and Rural Affairs, Beijing, China) [18]. The final body weight was recorded 2 h before slaughter. After slaughter, the head, skin, tail, and all internal organs except kidneys were removed; the carcass weight, loin eye muscle area, backfat thickness, meat weight, bone weight, meat color (L*, a*, b*, C*, and H°), and pH values were determined. Longissimus dorsi (LD) muscle between ribs 12 and 13 of the left carcass was taken for chemical and nutrient determination. Appropriate samples were stored in polyformaldehyde and liquid nitrogen for H&E staining and muscle fiber type determination.

### 2.1. Determination of pH Value

The pH values of the upper, middle, and lower parts of the LD muscle were measured by pH-meter (206, Tetu Instrument Co., Ltd., Shenzhen, China) at 45 min and 24 h after slaughter. The pH-meter was calibrated with a calibrated buffer solution before use (pH 4.00 and pH 6.86).

### 2.2. Determination of Meat Color

The lightness (L*), redness (a*), and yellowness (b*) values of LD muscle were measured by NR10QC portable chromaticity analyzer (3nh Technology Co. Ltd., Shenzhen, China) according to the instructions. The Chroma* (C*) and Hue* (H°) were calculated, and the average values were obtained by changing the position for three times.

### 2.3. Determination of Water Retention and Shear Force

Dripping loss: After slaughter, 100 g LD muscle was taken, weighed, hung with wire, packed into a plastic bag and sealed with air, placed at 4 °C for 24 h, and weighed and calculated. Cooking loss: The 150 g of LD muscle sample was taken; the fascia and attached fat were removed, weighed, and placed in a self-sealing bag, which was heated in a water bath for 30 min until the center temperature of the meat was 70 °C. The surface moisture of the meat was absorbed by filter paper, weighed, and calculated. Shear force: The meat samples with a central temperature of 0–4 °C were heated in a water bath at 80 °C so that the central temperature of meat samples reached 70 °C. After cooling, samples were taken along the direction of muscle fibers with a sampler with a diameter of 1.27 cm and immediately determined.

### 2.4. Determination of Nutrients in LD Muscle

Moisture: Take about 200 g LD muscle, remove fat and fascia, cut into pieces, and dry in a drying oven at 101–105 °C to a constant weight. Fat: Weigh fully mixed meat sample 5 g and transfer it to the filter paper cartridge. The filter paper cartridge is extracted by Soxhlet and the fat content is calculated. Protein content: Determination and calculation of protein content was performed with a Kjeldahl nitrogen analyzer (K9840, Shanghai Lijing Scientific Instrument Co. Ltd., Shanghai, China). Ash: After taking the beef sample, add Mg(CH_3_COO)_2_ solution (240 g/L) to moisten the sample completely. After 10 min, the sample is carbonized on the electric heating plate until smokeless, and then the sample is burned in the Muffle furnace at 550 °C to a constant weight.

### 2.5. Determination of Fatty Acids

Fatty acid analysis: The fatty acid spectrum was determined by Thermo Fisher Trace1310 ISQ gas chromatography-mass spectrometry (Thermo Fisher Scientific, Waltham, MA, USA) equipped with column TG-5MS. Heating procedure: The procedure is heating at 80 °C for 1 min at the rate of 10 °C/min, heating up to 200 °C, continue at the rate of 5 °C/min heating up to 250 °C, and finally at the rate of 2 °C/min rising to 270 °C, maintain for 3 min.

### 2.6. Determination of Amino Acids

Take the preserved muscle sample and grind it up. The samples were hydrolyzed with HCL at 110 °C for 22 h. Remove and cool, vacuum dry, and fill with nitrogen. Add the phenyl isothiocyanate derivative solution, derivate at room temperature for 30 min, and then apply the membrane to the machine. The qualitative determination of amino acids was performed by liquid chromatography with high-performance liquid chromatograph Agilent 1260 (Agilent Technologies Inc., Santa Clara, CA, USA), ultraviolet detector, and C18 column.

### 2.7. Determination of Inosine Acid and Guanylate Content

The sample was homogenized with 5% perchloric acid solution and centrifuged at 4 °C at 4000 r/min for 10 min. Adjust the pH of the supernatant with sodium hydroxide solution to 6.5, and pass the film to be tested. A Thermo Fisher U3000 high-performance liquid chromatograph (Thermo Fisher Scientific, Waltham, MA, USA) with Accucore C18 column (250 mm × 4.6 mm × 2.6 μm) was used to detect the contents of inosine and guanylate. Chromatographic conditions: mobile phase: triethylamine phosphate solution + methanol = 95 + 5; flow rate: 0.5 mL/min; UV detector: 254 nm.

### 2.8. Histological Analyses

A fixed muscle sample soaked in 4% paraformaldehyde solution was embedded in paraffin wax, sliced, mounted on a slide, and photographed on slides with a microscope. Use Caseviewer (version 2.4) to measure and calculate muscle fiber density and cross-sectional area.

### 2.9. Determination of Muscle Fiber Type

The procedure for detecting mRNA expression levels of *MyHC-I*, *MyHC-IIa*, *MyHC-IIx*, and *MyHC-IIb* followed the previously method with slight modification. The primers used are shown in Table 1, and set the *GAPDH* gene as the internal control. Gene expressions were calculated by the 2^−△△CT^ method.

### 2.10. Statistical Analysis Method

The values were expressed as mean ± standard deviation (M ± SD). The results were analyzed by one-way ANOVA and Duncan’s test with SPSS 20.0 software. The *p*-values of <0.05 were considered to be statistically significant.

## 3. Results

### 3.1. Comparison of Slaughter Performance

The carcass traits of the six breeds of cattle are shown in Table 2. The dressing percentage of Jinjiang cattle was significantly higher than other breeds (*p* < 0.05), and the dressing percentage of Guangfeng and Ji’an cattle and Xinfeng mountain buffalo was significantly higher than that of Xiajiang buffalo and Poyanghu buffalo (*p* < 0.05). Jinjiang cattle had the highest net meat rate and meat–bone ratio, which proved that Jinjiang cattle had the best meat production capacity, followed by Xinfeng mountain buffalo. Poyanghu buffalo had the worst meat production performance. The loin eye muscle area of yellow cattle breeds are bigger than that of buffalo breeds; Guangfeng cattle have the largest loin eye muscle area and Xiajiang buffalo the smallest (*p* < 0.05). Jinjiang cattle had the largest backfat thickness and Ji’an cattle had the smallest.

### 3.2. Comparison of Meat Quality

The meat quality of the six breeds is shown in Table 3. Guangfeng cattle have larger L*, a*, b*, and C* values among the yellow cattle breeds, followed by Ji’an cattle. Poyanghu buffalo had the largest L*, a*, and C* values, while Xiajiang buffalo had the largest b* and H° values. Yellow cattle breeds have larger L* and H° values and smaller a* values than buffalo breeds. In general, the meat of cattle is lighter and yellower, while buffalo is darker and redder. The fat color of Ji’an cattle is yellowish, and the fat color of other breeds is whiter. The pH_24h_ of experimental animals was less than 6.0, indicating that the stress before slaughter was low. The meat tenderness of Guangfeng cattle is obviously better than that of other breeds, and the tenderness of Poyanghu buffalo is second (*p* < 0.05). The drip loss and cooking loss of Jinjiang cattle were at the highest level, while the above indexes of Guangfeng cattle were the smallest (*p* < 0.05).

### 3.3. Comparison of the Nutritional Ingredients

The contents of conventional nutrients in the six breeds were at normal levels (Table 4). The moisture content of Guangfeng cattle was the lowest, and that of Poyanghu buffalo was the highest (*p* < 0.05). Compared with other breeds, the protein content of Guangfeng cattle is lower, but the IMF content is highest (*p* < 0.05). The IMF content of three yellow cattle breeds was higher than that of buffalo breeds, and the content of Xinfeng mountain buffalo was the lowest. There was no significant difference in ash content among the six cattle breeds.

### 3.4. Amino Acid Composition of Different Breeds

**Figure 2:**
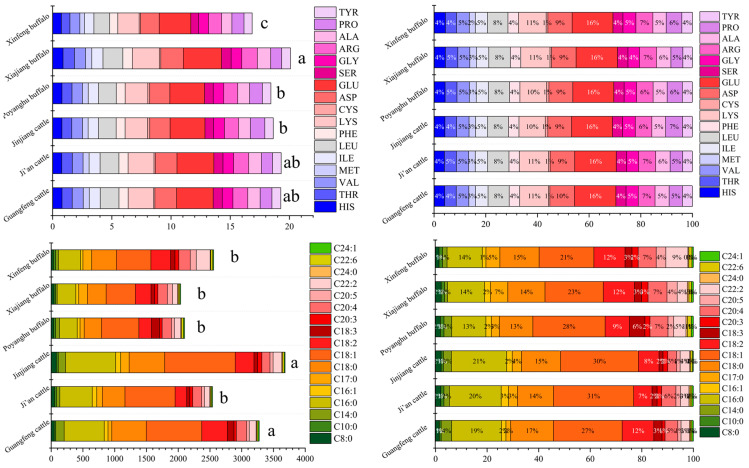
Comparison of differences in total amino acids and total fatty acids. The values were presented as mean ± SD (*n* = 10). For each measure, the difference in letters between the groups indicated significant differences by the letters a, b and c.

The amino acid composition of various breeds is shown in Table 5 and Figure 2, and the amino acid composition varies according to different breeds. The EAA/NEAA values of the six breeds were about 0.8, indicating that the amino acid composition was reasonable. Among them, Xinfeng mountain buffalo had the highest proportion of umami AA (Glutamic +Aspartic), sweet AA (Glycine + Alanine + Serine + Threonine + Proline), and proportion of essential amino acids and the best amino acid composition (*p* < 0.05), followed by Ji’an cattle.

### 3.5. Comparison of Fatty Acid Composition

The fatty acid composition of various breeds is shown in Table 6 and Figure 2, and the fatty acid composition of different varieties is quite different. The unsaturated fatty acid (UFA) content of Jinjiang cattle was the highest, followed by Guangfeng cattle. The UFA content of the Poyanghu buffalo was the highest in buffalo breeds, and that of the Xinfeng mountain buffalo was the lowest. The UFAs of buffalo are mainly polyunsaturated fatty acids (PUFAs), while the UFAs of cattle are mainly monounsaturated fatty acids (MUFAs). The ratio of omega 6 (n6) to omega 3 (n3) PUFAs has been recommended for health management. The n6:n3 ratio of buffalo is generally lower than that of yellow cattle, and the value of the Xiajiang buffalo is the lowest (*p* < 0.05).

### 3.6. Comparison of Flavor Nucleotides

**Figure 3:**
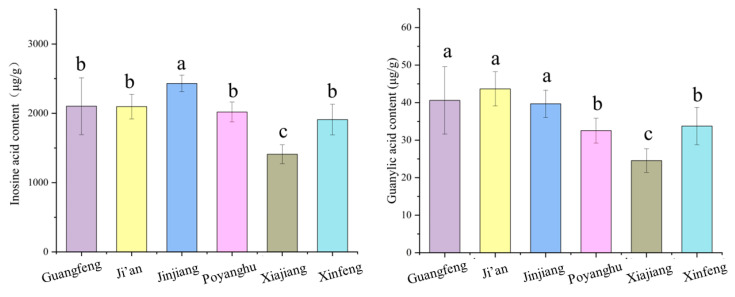
Comparison of differences in inosine and guanylate levels. The values were presented as mean ± SD (*n* = 10). For each measure, the difference in letters between the groups indicated significant differences by the letters a, b and c.

The inosine and guanylic acid contents of the six breeds are shown in Figure 3. The inosine acid content of Jinjiang beef was the highest, while the content in Xiajiang buffalo beef was the lowest. The level of guanylic acid in yellow cattle breeds was higher than that of buffalo breeds (*p* < 0.05). The levels of two flavoring nucleotides in Xiajiang buffalo were the lowest, and the levels in Jinjiang cattle were highest (*p* < 0.05).

### 3.7. Comparison of Muscle Fiber Morphology and Muscle Bundle Properties

**Figure 4:**
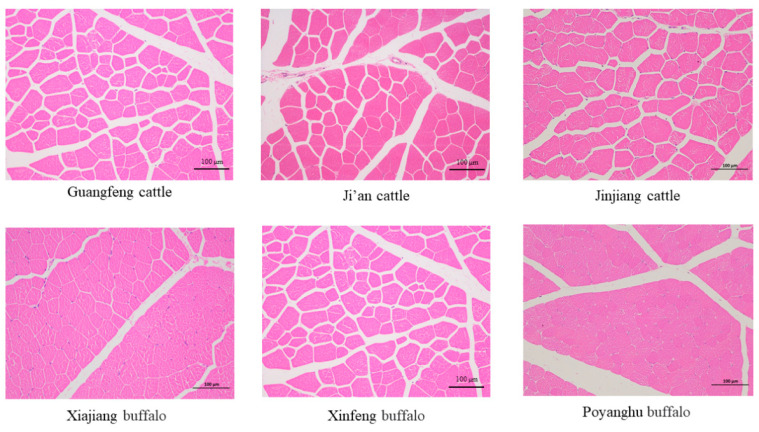
Histological sections of LD muscle of different breeds of cattle.

As shown in Table 7 and Figure 4, the diameter and area of muscle fibers of yellow cattle are generally larger than that of buffalo, and the muscle fiber density is generally smaller than that of buffalo. The diameter and area of muscle fibers of Guangfeng cattle were the smallest among yellow cattle breeds, and that of Xiajiang buffalo was the smallest among all breeds.

### 3.8. Comparison of Muscle Fiber

**Figure 5:**
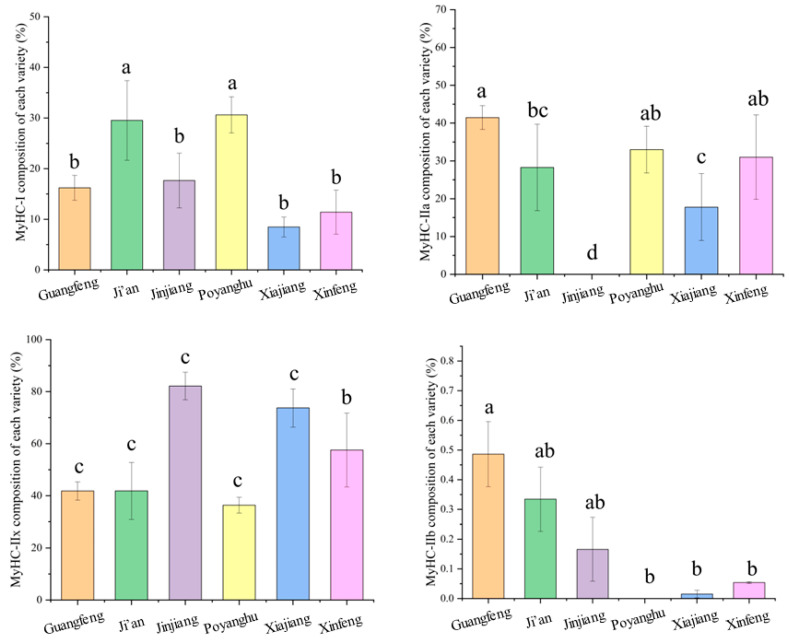
Comparison of differences in muscle fiber type. The values were presented as mean ±SD (n = 6). For each measure, the difference in letters between the groups indicated significant differences by the letters a, b, c, and d (*p* < 0.05).

Myosin heavy chain-Ⅰ (*MyHC-Ⅰ*), *MyHC-Ⅱa*, and *MyHC-Ⅱx* were the main types of muscle fibers in the six breeds (Figure 5). The *MyHC-Ⅰ* and *MyHC-Ⅱa* muscle fibers of Guangfeng cattle, Ji’an cattle, and Poyanghu buffalo accounted for about 55%, the *MyHC-Ⅱx* muscle fibers of Jinjiang cattle and Xiajiang buffalo accounted for about 75–80%, and the *MyHC-Ⅱx* muscle fibers of Xinfeng mountain buffalo accounted for about 60%.

### 3.9. Correlation Analysis between Varieties and Characters

**Figure 6:**
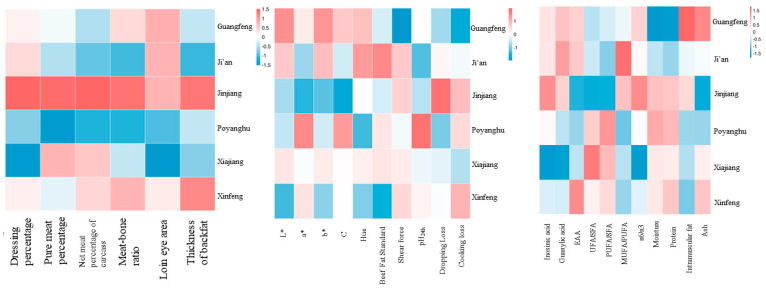
Correlation analysis of nutritional components of different breeds.

The correlation heat map of each breed and each performance is shown in Figure 6. In terms of carcass traits, Jinjiang cattle had the best slaughter performance, followed by Xinfeng mountain buffalo. The meat quality of Guangfeng cattle was the best, and the nutritional value of Xinfeng mountain buffalo is higher than other breeds. Compared with buffalo beef, the beef of yellow cattle breeds may have a better flavor.

## 4. Discussion

Dressing percentage is one of the important indexes to reflect the output of livestock and poultry meat, and a higher dressing percentage means a higher meat production capacity of the livestock. At the same time, the dressing percentage is also one of the important factors in determining the commercial value of the carcass [19]. Breed difference may be one of the main reasons for the differences in dressing percentage. The results of this study showed that the dressing percentage of local breeds of cattle in Jiangxi was slightly higher than that of buffalo, and the dressing percentage of Jinjiang cattle and Ji’an cattle was better at 53.82% and 50.49%, respectively. The dressing percentage for *Japanese black cattle* and *Simonsit × Angus* cattle were reported to be 60.1% and 61.6%, respectively, and about 55% for grass-fed *Angus* cattle [20,21]. Previous studies have shown that higher fat content in carcasses leads to a higher dressing percentage [22,23]. Compared with the world-famous cattle breeds, the dressing percentage of local cattle in Jiangxi is slightly lower, possibly due to the breed differences and the lack of a rigorous fattening process. Loin eye muscle area has been proven to be significantly positively correlated with net meat weight and dressing percentage of cattle, which is one of the important indicators to measure meat quality in livestock production and has great significance in animal genetics and breeding [24]. The loin eye muscle area is related to fiber diameter and fiber length, as both increase muscle circumference [25]. Furthermore, the size of the loin eye muscle area is important in meat production, as the ribs made from this part are considered superior and are the most expensive in specialized restaurants and markets [26]. The results of this study were similar to previous studies, with breeds with large loin eye muscle areas having a higher dressing percentage. The loin eye muscle area of cattle breeds was larger than that of buffalo breeds, which was consistent with the measurement results of muscle fiber diameter and area, indicating that the carcass of cattle may have higher commercial value. The reason for the low backfat thickness of the animals used in this study may be related to the feeding method of the animals used for grazing without strict fattening technology, and it also implies that the lean meat rate of local cattle in Jiangxi is higher, which can better meet the increasing demand for lean beef [27].

Color, tenderness, flavor, and juiciness are important attributes that determine the sensory properties of meat, while the chemical composition and physical properties determine the quality of meat [28]. The color is an important standard to measure meat quality traits. It is the first factor seen by consumers that affects purchasing decisions and is used as an indicator of freshness and health [29]. In general, consumers prefer bright red beef to purple or brown beef [30]. The color of meat depends on breed, age, and muscle type, and the difference in color is mainly affected by myoglobin content, pH, and physicochemical status [31,32]. It has been reported that the higher the myoglobin concentration, the darker the color of the meat [28]. Beef with high *MyHC-Ⅰ* and *MyHC-Ⅱa* muscle fiber content usually has a higher a* value [33]. The pH value will affect the activity and oxidation rate of enzymes, and a higher pH value will stabilize the color of beef [34,35]. The increase in IMF will increase the content of myoglobin, which makes the color of the meat redder by providing more oxygen requirements. In addition, grazing may also darken the flesh [36]. In this study, the a* value of buffalo cattle was higher than that of cattle (except Guangfeng cattle), and the difference in meat color was significantly related to the variety. Poyanghu buffalo had the highest a* value, which may be due to its high *MyHC-Ⅰ* fiber content. The a* value of Guangfeng cattle may be related to the higher content of IMF and *MyHC-Ⅱa* muscle fiber. The pH is an important index to judge meat quality, which is closely related to meat color, tenderness, juiciness, and flavor. Pre-slaughter stress causes muscle glycogen depletion, which ultimately leads to an increase in pH, resulting in dark-cutting beef that has reduced tenderness, juiciness, and flavor [37]. However, an excessive drop in pH after death reduces the water-retaining capacity of the muscles [38]. The final pH value of different breeds of cattle in this study was less than 6.0, indicating that the glycogen level in the muscle was high enough to establish a good lactic acid level, thereby extending the shelf life of the meat and reducing the probability of dark-cutting [39,40,41].

The eating characteristic is the main meat characteristic, which is mainly affected by intramuscular fat content, tenderness, juiciness, and flavor [42]. Tenderness proved to be the most important factor in consumers’ perception of meat quality [43]. A study of consumers in the United States determined that when the Warner–Bratzler shear force value was less than or equal to 4.1 kg, the acceptances reached 98% [44]. The tenderness of beef is affected not only by breed, sex, age, and maturity, but also by the final pH value, muscle fiber characteristics, and intermuscular fat content. It has been reported that the tenderness of beef decreased as pH increased from 5.5 to about 6.0, and increased when pH increased from 6.0 to 7.0. Beef in the normal pH range (pH 5.50–5.80) and the higher pH range (pH > 6.1) showed enhanced tenderness [45,46]. The pH_24h_ of each breed of cattle in this study was between 5.98 and 5.57, indicating that the effect of pH on beef was in the appropriate range. Muscle fiber characteristics (fiber number, fiber cross-sectional area, and type) are important factors affecting meat quality. Compared with *MyHC-Ⅰ* muscle fiber, *MyHC-Ⅱ* muscle fiber has higher glycogen levels, a stronger glycolysis ability, and is more tough. Among cattle and sheep, *MyHC-Ⅰ* was reported to have the smallest fiber cross-sectional area; *MyHC-Ⅱb* was larger, and *MyHC-Ⅱa* and *MyHC-Ⅱx* were reported to have medium sizes. The smaller the diameter of muscle fibers, the larger the density, the smaller the shear force, and the better the tenderness of meat. Essen-Gustavsson (1994) reported that lipids were mainly stored in *MyHC-Ⅰ* fibers and some *MyHC-Ⅱa* fibers, suggesting that meat with higher types of muscle fibers had better tenderness [47]. The expansion of marbled fat cells can force muscle bundles apart, opening the muscle structure, and muscles with marbled fat concentrations higher than 30 mg/g produce the best tenderness [48,49]. The results of this study showed that the diameter and area of muscle fibers of cattle were generally larger than that of buffalo, while the density of muscle fibers was smaller than that of buffalo, which may be the reason the shear-force of buffalo breeds was lower than that of Ji’an cattle and Jinjiang cattle. The optimum tenderness of Guangfeng cattle may be the result of the combination of the highest intermuscular fat content, the higher level of *MyHC-Ⅱa* and *MyHC-Ⅰ* fibers, the lower level of *MyHC-Ⅱx* fibers, and the relatively small muscle fiber diameter.

The juiciness of meat is a very important edible characteristic, and depends on water and fat levels of meat. Factors that affect the water retention capacity and fat content of meat may affect meat juiciness, such as final pH, fat content, curing method, cooking method, and doneness [43,50]. The cooking loss, drip loss, and compressed-based methods are commonly used to quantify expressible moisture in meat. Cooking losses have been reported to explain 60–80% of the difference in juiciness [51]. The IMF is often referred to as marbled fat composed mainly of neutral lipids [52] and could prevent meat from drying out during cooking [49]. In addition, the contraction of meat is related to the degeneration of myofibrillar fibers and connective tissue proteins in the muscle structure during cooking [53,54]. High *MyHC-Ⅰ* fiber content is more conducive to meat juiciness and flavor, while high *MyHC-Ⅱb* fiber content is often associated with tougher meat quality [55]. In this study, there was no obvious rule in the water retention ability of buffalo and cattle. Guangfeng cattle had the best water retention ability, followed by Xiajiang buffalo. Guangfeng beef shows the best water retention ability, possibly because Guangfeng beef has a high intermuscular fat content, which also means that Guangfeng beef has the best juiciness.

The moisture in the muscle has an important impact on the color, texture, and surface appearance of the meat, and the moisture content of each variety in this experiment is about 75%, which is at a normal level. The crude protein content of the 6 Jiangxi local breeds ranged from 18.46% to 22.23%, which was significantly higher than of *Angus* steers (13%), suggesting that Jiangxi local breeds could provide higher protein nutritional value [56]. Saturated fatty acid (SFA), monounsaturated fatty acid (MUFA), and polyunsaturated fatty acid (PUFA) are common fatty acids in meat products, and it is generally believed that a higher intake of unsaturated fatty acid (UFA) is beneficial to body health and can significantly reduce the risk of cardiovascular disease [57]. The fat content and fatty acid composition of meat have become a point that has aroused consumers’ attention and affected consumers’ purchase [58]. This is especially true of grazed beef products, which contain higher proportions of omega-3 and long-chain fatty acids EPA + DHA [59]. The n-3 and n-6 PUFAs have the capacity to promote human growth and development, safeguard maternal and infant health, and protect cognitive. The World Health Organization recommends that various dietary fat components account for <15–30%, <10%, <5–8%, <1–2%, and <1% of total energy intake of total fat, SFA, n-6 PUFA, n-3 PUFA, and trans fatty acids, respectively [60]. Achieving an n-6:n-3 ratio between 1:1 and 4:1 throughout the diet is considered beneficial for human health. The average percentage of intermuscular fat in mature beef has been reported to be 0.45–0.48 SFA, 0.35–0.45 MUFA, and up to 0.05 PUFA. The SFA ratio in this study was 0.41–0.39, the MUFA ratio was 0.28–0.25, and the PUFA ratio was 0.33–0.34, probably because the local cattle used in this study were grazing; the experimental animals had a lot of lean meat [61]. Compared to reported fatty acid data from *Angus*, *Brahmin*, and *Romenu* cattle, the six breeds we studied showed relatively low SFA and high UFA levels [62]. The UFA levels in buffalo are generally higher than those in cattle, and the UFAs in cattle are mainly MUFA, while those in buffalo are mainly PUFA. The n6:n3 of buffalo is smaller than that of cattle, and both are less than 4.0, suggesting that the fatty acid composition of buffalo meat may be more beneficial to human health. Amino acids are key indicators of nutritional quality and the main precursors of specific flavor substances produced in meat through the Maillard reaction and sugar reduction degradation [63]. According to the FAO/WHO model standards, the quality of the protein EAA/TAA should be about 40%, and EAA/NEAA should be more than 60% [64]. The daily requirements of EAAs and NEAAs for adult males are 0.18 g/kg and 0.48 g/kg, which correspond to EAAs/NEAAs = 37.5% and EAAs/TAAs = 27.3%, respectively [65]. The EAA ratio of Jiangxi cattle was basically the same as that of *Aberdeen Angus* and *Hungarian Simmental* cattle reported previously (51% and 47%, respectively) [66,67]. The EAA/TAA of the 6 breeds were all greater than 40% and the EAA/NEAA was about 80%, indicating that Jiangxi cattle were the source of high-quality protein.

Many factors play an important role in the development of beef flavor, including marbling level, animal diet, age, and genetics/cattle type. The main precursors of beef flavor can be divided into the following two categories: water-soluble components (amino acids, peptides, carbohydrates, nucleotides, thiamine, etc.) and lipids [68]. The complex fatty acids in meat melt between 25 °C and 50 °C, with SFA melting at higher temperatures and PUFA melting at lower temperatures [69]. The vast majority of studies believe that increasing the marbling fraction of beef and increasing the MUFA content of beef will improve the flavor performance of beef [70]. It has been reported that beef from wagyu cattle is believed to contain a higher proportion of MUFAs, especially C18:1, which is associated with improved beef flavor traits [64,71]. The presence of PUFAs, particularly the n-3 PUFAs, has been proven to have a negative impact on beef flavor [72]. In this study, the MUFA and C18:1 content of Guangfeng cattle, Ji’an cattle, and Jinjiang cattle were higher than that of buffalo, and the Jinjiang beef content was the highest. In addition, fat binds to other components such as amino acids and proteins when heated, becoming one of the precursors of flavor [52]. Ma et al. (2017) reported that an increase in the number of free amino acids correlates with the degree of protein hydrolysis, which indicates a better flavor of meat [73]. Umami, which responds to substances such as flavorful amino acids and flavorful nucleotides (inosine and guanylate), can enhance appetite and satiety, improve eating disorders, and correct impaired taste. In terms of muscle fiber types, oxidized fiber, and intermediate fiber types have a higher crude fat content and a higher proportion of phospholipids, both of which have been shown to contribute significantly to beef flavor [74]. The three varieties of cattle have a higher content of umami amino acids and taste nucleotides (inosine and guanylate), suggesting that yellow beef may have a better flavor. Many researchers have shown that the taste of beef is gradually not accepted by consumers as the pH level increases or the degree of darkening increases, and the upper loin steak with pH > 6.0 has a lower overall favorability [75,76]. In this study, the pH_24h_ of six breeds of cattle was lower than 6.0, the flavor was normal, and no black-cutting meat was produced.

## 5. Conclusions

In conclusion, this study provides a comprehensive database of carcass characteristics and meat quality of local cattle breeds in Jiangxi. Among the local breeds in Jiangxi, Jinjiang cattle had the best slaughtering performance. Guangfeng cattle have the characteristics of good tenderness, high water retention and high fat content. Xiajiang buffalo had the highest proportion of UFA and the lowest n6:n3 value. Consumers who are looking for better meat quality can choose Guangfeng beef, and consumers who are looking for health benefits can prioritize buffalo beef, especially Xiajiang buffalo beef. To achieve better economic results, internationally recognized breeds (Simmental, Charolais) are selected to improve the carcass traits of local breeds. However, while introducing foreign varieties, it is also necessary to protect local resources and highlight the advantages of local resources.

## Figures and Tables

**Table 1:** Primers used in this experiment.

Gene	Primers	Product Size (bp)	Accession NO.
*MyHC-I*	Forward: 5′-AACCTGTCCAAGTTCCGCAAG-3′ Reverse: 3′-AGATGTGGCACGGCTACTCCT-3′	148	NM_174727
*MyHC-IIa*	Forward: 5′-TGGAGCGGATGAAGAAGAACA-3′ Reverse: 3′-GCTTCTGCTCACTCTCTACCTCTC-3′	159	NM_001166227
*MyHC-IIb*	Forward: 5′-AGTGCTATCCCAGAGGGTCAGT-3′ Reverse: 3′-AGCTTTTCATCTCGCATCTCCT-3′	161	AY227972
*MyHC-IIx*	Forward: 5′-AGTGCTATCCCAGAGGGTCAGT-3′ Reverse: 3′-AGCTTTTCATCTCGCATCTCCT-3′	226	NM_174117
*GAPDH*	Forward: 5′-AGGGCTGCTTTTAATTCTGGC-3′ Reverse: 5′-TGACTGTGCCGTTGAACTTGC-3′	124	NM_001034034

**Table 2:** Comparison of differences in carcass characteristics.

	Species	Guangfeng Cattle	Ji’an Cattle	Jinjiang Cattle	Poyanghu Buffalo	Xiajiang Buffalo	Xinfeng Mountain Buffalo
Index							
Dressing percentage (%)		49.69 ± 1.41 b	50.49 ± 1.85 b	53.82 ± 1.63 a	47.2 ± 1.62 c	44.88 ± 1.96 d	49.78 ± 1.55 b
Net meat percentage (%)		37.49 ± 1.22 b	36.36 ± 1.91 b	41.98 ± 1.51 a	33.36 ± 1.36 c	33.97 ± 1.62 c	37.27 ± 1.68 b
Meat–bone ratio		3.96 ± 0.43 bc	3.24 ± 0.69 d	4.49 ± 0.47 a	3.20 ± 0.32 d	3.58 ± 0.26 cd	4.16 ± 0.51 b
Loin eye muscle area (cm^2^)		62.40 ± 7.27 a	62.2 ± 9.85 a	61.8 ± 12.73 a	42.1 ± 4.87 b	36.3 ± 8.05 b	55.50 ± 8.57 a
Thickness of backfat (mm)		0.20 ± 0.11 bc	0.16 ± 0.08 c	0.30 ± 0.10 a	0.20 ± 0.07 bc	0.18 ± 0.05 c	0.29 ± 0.15 ab

Note: The values were presented as mean ± SD (*n* = 10). For each measure, the difference in letters between the groups indicated significant differences by the letters a, b, c, and d (*p* < 0.05).

**Table 3:** Comparison of differences in meat quality.

	Species	Guangfeng Cattle	Ji’an Cattle	Jinjiang Cattle	Poyanghu Buffalo	Xiajiang Buffalo	Xinfeng Mountain Buffalo
Index							
L*		31.10 ± 1.88 a	30.35 ± 1.71 ab	28.97 ± 1.94 cd	29.16 ± 1.68 bcd	29.99 ± 1.51 abc	28.33 ± 1.45 d
a*		10.98 ± 1.46 b	10.07 ± 1.69 bc	9.53 ± 1.66 c	12.08 ± 1.71 a	10.71 ± 1.33 b	11.07 ± 1.17 b
b*		7.22 ± 1.19 a	6.92 ± 1.31 ab	5.76 ± 1.48 d	6.24 ± 1.22 bcd	6.60 ± 1.03 bcd	5.95 ± 0.84 cd
C*		13.16 ± 1.75 ab	12.25 ± 1.97 b	11.18 ± 1.96 c	13.63 ± 1.88 a	12.60 ± 1.52 ab	12.60 ± 1.16 ab
H°		33.25 ± 3.05 a	34.52 ± 4.19 ab	31.05 ± 5.51 bc	27.34 ± 3.87 d	31.65 ± 3.31 ab	28.31 ± 4.01 cd
Beef fat standard		3.30 ± 0.90 bc	4.10 ± 0.54 a	3.30 ± 0.64 bc	3.60 ± 0.66 ab	3.60 ± 0.49 ab	2.80 ± 0.40 c
Shear force (kg/cm^2^)		7.50 ± 1.43 b	10.30 ± 1.79 a	10.22 ± 1.31 a	9.49 ± 1.46 a	9.87 ± 2.39 a	10.13 ± 1.53 a
pH_45min_		6.91 ± 0.57 a	6.71 ± 0.14 b	6.61 ± 0.28 b	6.65 ± 0.21 b	6.53 ± 0.19 b	6.64 ± 0.10 b
pH_24h_		5.76 ± 0.30 b	5.57 ± 0.07 c	5.67 ± 0.17 bc	5.98 ± 0.08 a	5.74 ± 0.16 bc	5.78 ± 0.27 b
Dripping loss (%)		1.41 ± 0.29 b	1.69 ± 0.56 b	2.26 ± 0.64 a	1.32 ± 0.29 b	1.58 ± 0.28 b	1.65 ± 0.27 b
Cooking loss (%)		19.29 ± 4.16 c	23.13 ± 3.37 ab	25.56 ± 2.83 a	24.63 ± 2.06 ab	21.81 ± 2.36 b	25.97 ± 3.87 a

Note: The values were presented as mean ± SD (*n* = 10). For each measure, the difference in letters between the groups indicated significant differences by the letters a, b, c, and d (*p* < 0.05).

**Table 4:** Comparison of differences in nutritional ingredients.

	Species	Guangfeng Cattle	Ji’an Cattle	Jinjiang Cattle	Poyanghu Buffalo	Xiajiang Buffalo	Xinfeng Mountain Buffalo
Index							
Moisture (%)		73.63 ± 2.29 b	75.07 ± 2.46 ab	76.41 ± 0.98 a	76.69 ± 0.85 a	75.88 ± 1.16 ab	75.89 ± 4.92 ab
Protein (%)		18.46 ± 1.70 b	19.89 ± 2.33 ab	21.96 ± 1.03 a	22.23 ± 1.12 a	21.30 ± 2.55 a	21.95 ± 4.73 a
Intramuscular fat (%)		1.87 ± 0.46 a	0.91 ± 0.41 bc	1.20 ± 0.65 b	0.62 ± 0.29 c	0.69 ± 0.25 c	0.51 ± 0.21 c
Ash (%)		1.06 ± 0.11 a	1.00 ± 0.08 a	0.94 ± 0.04 a	0.97 ± 0.08 a	1.01 ± 0.07 a	1.03 ± 0.22 a

Note: The values were presented as mean ± SD (*n* = 10). For each measure, the difference in letters between the groups indicated significant differences by the letters a, b and c. (*p* < 0.05).

**Table 5:** Comparison of differences in amino acid.

	Species	Guangfeng Cattle	Ji’an Cattle	Jinjiang Cattle	Poyanghu Buffalo	Xiajiang Buffalo	Xinfeng Mountain Buffalo
g/100 g							
Aspartate		1.84 ± 0.12 ab	1.83 ± 0.17 ab	1.72 ± 0.11 b	1.58 ± 0.08 c	1.71 ± 0.09 b	1.91 ± 0.16 a
Glutamate		3.09 ± 0.21 ab	3.11 ± 0.27 ab	2.94 ± 0.20 b	2.66 ± 0.15 c	2.93 ± 0.11 b	3.22 ± 0.25 a
Serine		0.76 ± 0.05 b	0.78 ± 0.07 ab	0.73 ± 0.04 b	0.65 ± 0.04 c	0.74 ± 0.02 c	0.82 ± 0.06 a
Glycine		0.91 ± 0.08 a	0.89 ± 0.08 a	0.92 ± 0.08 a	0.85 ± 0.06 a	0.89 ± 0.06 a	0.90 ± 0.08 a
Histidine		0.79 ± 0.06 b	0.77 ± 0.06 b	0.78 ± 0.06 b	0.74 ± 0.04 b	0.79 ± 0.03 b	0.88 ± 0.06 a
Arginine		1.25 ± 0.09 ab	1.27 ± 0.12 ab	1.19 ± 0.08 bc	1.10 ± 0.06 c	1.18 ± 0.06 bc	1.32 ± 0.12 a
Threonine		0.86 ± 0.06 b	0.90 ± 0.07 b	0.83 ± 0.05 b	0.72 ± 0.05 c	0.85 ± 0.03 b	0.97 ± 0.08 a
Alanine		1.05 ± 0.07 ab	1.09 ± 0.09 a	1.00 ± 0.06 b	0.87 ± 0.07 c	1.01 ± 0.05 b	1.12 ± 0.12 a
Proline		0.99 ± 0.11 bc	0.93 ± 0.06 c	1.22 ± 0.07 a	1.02 ± 0.21 bc	1.08 ± 0.10 b	0.93 ± 0.04 c
Tyrosine		0.75 ± 0.05 a	0.74 ± 0.06 ab	0.72 ± 0.04 ab	0.69 ± 0.05 b	0.72 ± 0.02 cb	0.75 ± 0.04 a
Valine		0.94 ± 0.06 a	0.91 ± 0.06 a	0.90 ± 0.05 ab	0.84 ± 0.06 b	0.88 ± 0.04 cb	0.93 ± 0.07 a
Methionine		0.50 ± 0.03 ab	0.51 ± 0.04 ab	0.48 ± 0.03 ab	0.39 ± 0.05 c	0.49 ± 0.03 cb	0.53 ± 0.05 a
Isoleucine		0.93 ± 0.06 a	0.93 ± 0.07 a	0.87 ± 0.05 b	0.79 ± 0.04 c	0.86 ± 0.03 b	0.95 ± 0.09 a
Leucine		1.58 ± 0.10 ab	1.59 ± 0.12 ab	1.50 ± 0.09 b	1.33 ± 0.07 c	1.51 ± 0.05 b	1.67 ± 0.15 a
Cysteine		0.10 ± 0.03 c	0.11 ± 0.02 c	0.19 ± 0.03 ab	0.11 ± 0.05 c	0.16 ± 0.05 ab	0.12 ± 0.04 bc
Phenylalanine		0.78 ± 0.05 ab	0.78 ± 0.06 ab	0.76 ± 0.04 b	0.69 ± 0.04 c	0.75 ± 0.02 b	0.81 ± 0.06 a
Lysine		2.15 ± 0.16 a	2.19 ± 0.21 a	1.89 ± 0.15 b	1.81 ± 0.09 b	1.91 ± 0.11 b	2.27 ± 0.24 a
Umami AA		4.92 ± 0.33 ab	4.94 ± 0.43 ab	4.66 ± 0.31 b	4.25 ± 0.23 c	4.63 ± 0.18 b	5.13 ± 0.43 a
Sweet AA		4.57 ± 0.33 a	4.58 ± 0.34 a	4.70 ± 0.27 a	4.11 ± 0.35 b	4.56 ± 0.19 a	4.73 ± 0.33 a
Bitter AA		7.52 ± 0.47 ab	7.48 ± 0.58 ab	7.19 ± 0.41 b	6.58 ± 0.36 c	7.18 ± 0.25 b	7.84 ± 0.64 a
EAA (%)		44.29 ± 0.38 b	44.34 ± 0.27 b	43.01 ± 0.34 d	43.39 ± 0.42 cd	43.57 ± 0.34 c	43.31 ± 0.62 a
NEAA (%)		55.17 ± 0.33 c	55.07 ± 0.32 c	55.99 ± 0.36 a	55.95 ± 0.25 a	55.56 ± 0.30 b	54.55 ± 0.65 d
EAA/NEAA		0.80 ± 0.01 b	0.81 ± 0.02 b	0.77 ± 0.01 d	0.78 ± 0.01 cd	0.78 ± 0.01 c	0.82 ± 0.02 a

Note: The values were presented as mean ± SD (*n* = 10). For each measure, the difference in letters between the groups indicated significant differences by the letters a, b, c, and d (*p* < 0.05). EAA: Essential amino acids (Lysine + Methionine + Threonine + Leucine +Isoleucine + Valine + Phenylalanine + Histidine). Umami AA: (Glutamic +Aspartic). Sweet AA: (Glycine + Alanine + Serine + Threonine + Proline). Bitter AA: (Arginine + Histidine + Leucine + Isoleucine + Methionine + Phenylalanine + Tyrosine + Valine).

**Table 6:** Comparison of differences in fat acid.

	Species	Guangfeng Cattle	Ji’an Cattle	Jinjiang Cattle	Poyanghu Buffalo	Xiajiang Buffalo	Xinfeng Mountain Buffalo
100 g/kg							
C8:0		47.10 ± 14.01 b	52.25 ± 25.67 b	79.92 ± 26.62 a	34.67 ± 16.61 b	38.76 ± 13.02 b	44.66 ± 15.20 b
C10:0		23.59 ± 6.17 b	29.98 ± 9.53 ab	30.96 ± 9.94 ab	34.37 ± 15.47 a	29.53 ± 4.67 ab	25.56 ± 7.45 ab
C14:0		133.81 ± 142.78 a	53.67 ± 22.81 bc	115.06 ± 36.83 ab	44.46 ± 18.16 c	63.39 ± 26.68 bc	24.01 ± 9.65 c
C16:0		631.09 ± 236.12 ab	510.27 ± 208.59 bc	788.90 ± 315.35 a	349.89 ± 92.57 cd	278.09 ± 65.37 d	293.12 ± 37.71 d
C16:1		58.42 ± 25.90 abc	71.67 ± 40.69 ab	79.68 ± 35.72 a	36.12 ± 7.68 c	44.46 ± 9.00 bc	46.18 ± 35.68 bc
C17:0		59.80 ± 28.57 c	88.48 ± 29.06 b	132.66 ± 26.83 a	138.06 ± 41.84 a	65.63 ± 6.58 bc	137.54 ± 27.44 a
C18:0		543.37 ± 160.51 a	355.03 ± 136.21 b	559.49 ± 196.47 a	389.76 ± 124.24 b	272.73 ± 69.81 b	294.02 ± 114.83 b
C18:1n9c		873.61 ± 437.44 ab	789.80 ± 358.35 abc	1110.85 ± 548.96 a	543.05 ± 319.91 bc	588.26 ± 203.17 bc	460.55 ± 119.41 c
C18:2n6c		402.57 ± 114.11 a	181.24 ± 78.40 c	294.31 ± 79.08 b	309.03 ± 36.40 b	198.70 ± 31.28 c	246.53 ± 65.11 bc
C18:3n3		104.32 ± 25.62 b	50.01 ± 18.32 c	57.43 ± 23.28 c	68.82 ± 12.22 c	125.51 ± 14.22 a	55.96 ± 18.04 c
C20:3n6		37.99 ± 15.61 c	45.69 ± 23.90 bc	67.90 ± 30.47 a	61.46 ± 16.37 ab	41.72 ± 16.27 bc	51.55 ± 22.74 abc
C20:4n6		161.87 ± 38.56 ab	139.77 ± 55.81 ab	127.73 ± 37.98 b	179.28 ± 53.76 a	146.54 ± 19.83 ab	149.59 ± 50.14 ab
C20:5n3		41.22 ± 14.63 b	44.53 ± 20.78 b	49.02 ± 16.06 b	95.63 ± 15.99 a	46.14 ± 21.47 b	80.78 ± 21.16 a
C22:2n6		108.42 ± 49.65 b	84.03 ± 48.60 b	136.86 ± 68.07 b	218.08 ± 89.49 a	100.43 ± 19.15 b	79.79 ± 29.66 b
C22:6n3		18.25 ± 11.27 b	16.91 ± 10.57 b	26.04 ± 11.62 ab	31.23 ± 11.19 a	30.74 ± 13.60 a	20.27 ± 11.41 ab
C24:0		10.36 ± 3.46 a	11.45 ± 5.92 a	12.55 ± 3.83 a	8.93 ± 3.69 a	12.29 ± 5.72 a	12.77 ± 4.63 a
C24:1n9		19.94 ± 13.31 a	15.19 ± 6.12 a	13.67 ± 11.93 a	14.32 ± 6.79 a	17.10 ± 9.15 a	14.84 ± 8.66 a
SFA		1449.12 ± 481.38 a	1101.13 ± 311.66 b	1719.54 ± 522.87 a	1000.14 ± 196.94 b	760.42 ± 125.95 b	831.68 ± 131.33 b
UFA		1826.61 ± 373.31 ab	1438.64 ± 221.49 cd	1963.49 ± 580.01 a	1557.02 ± 219.46 bc	1339.60 ± 224.76 cd	1206.04 ± 90.63 d
MUFA		951.97 ± 454.21 ab	876.66 ± 383.62 abc	1204.20 ± 581.54 a	593.49 ± 321.57 bc	649.82 ± 201.54 bc	521.57 ± 131.99 c
PUFA		874.64 ± 123.47 ab	562.18 ± 195.21 d	759.29 ± 105.93 bc	963.53 ± 150.57 a	689.78 ± 65.40 cd	684.47 ± 127.40 cd
UFA/SFA		1.34 ± 0.26 c	1.37 ± 0.25 c	1.15 ± 0.09 d	1.57 ± 0.12 b	1.77 ± 0.13 a	1.48 ± 0.23 bc
PUFA/SFA		0.70 ± 0.32 bc	0.59 ± 0.32 c	0.48 ± 0.15 c	1.01 ± 0.27 a	0.92 ± 0.11 ab	0.85 ± 0.21 ab
MUFA/PUFA		1.17 ± 0.70 b	2.16 ± 1.85 a	1.63 ± 0.91 ab	0.68 ± 0.51 b	0.94 ± 0.28 b	0.82 ± 0.33 b
PUFA n = 3		163.79 ± 22.55 b	111.45 ± 31.80 d	132.49 ± 28.52 cd	195.68 ± 23.29 a	202.39 ± 30.72 a	157.01 ± 35.50 bc
PUFA n = 6		710.85 ± 111.64 ab	450.73 ± 167.37 d	626.80 ± 115.02 bc	767.85 ± 135.31 a	487.39 ± 55.39 d	527.46 ± 132.38 cd
n6:n3 ratio		4.38 ± 0.70 a	3.99 ± 0.59 a	5.11 ± 0.89 a	3.93 ± 0.85 a	2.46 ± 0.92 b	3.72 ± 0.85 a

Note: The values were presented as mean ± SD (*n* = 10). For each measure, the difference in letters between the groups indicated significant differences by the letters a, b, c, and d (*p* < 0.05).

**Table 7:** Comparison of differences in muscle fiber characteristics.

	Species	Guangfeng Cattle	Ji’an Cattle	Jinjiang Yellow Cattle	Poyanghu Buffalo	Xiajiang Buffalo	Xinfeng Mountain Buffalo
Index							
Fiber diameter (μm)		47.42 ± 5.73 a	49.55 ± 5.52 a	50.02 ± 2.92 a	43.17 ± 5.73 ab	35.47 ± 5.24 c	38.42 ± 3.48 bc
Fiber cross-sectional area (μm^2^)		2333.53 ± 605.48 a	2546.02 ± 553.01 a	2537.49 ± 328.52 a	1897.01 ± 503.11 ab	1302.31 ± 397.58 b	1594.60 ± 358.18 b
Fiber density (N/mm^2^)		324.09 ± 22.10 bc	412.32 ± 79.30 bc	354.83 ± 50.01 c	535.74 ± 112.57 a	533.29 ± 102.10 a	488.50 ± 84.34 ab

Note: The values were presented as mean ± SD (*n* = 6). For each measure, the difference in letters between the groups indicated significant differences by the letters a, b and c. (*p* < 0.05).

## Data Availability

The raw data supporting the conclusions of this article will be made available by the authors on request.

## References

[1] Zhou G.H., Liu L., Xiu X.L., Jian H.M., Wang L.Z., Sun B.Z., Tong B.S. (2001). Productivity and carcass characteristics of pure and crossbred Chinese Yellow Cattle. Meat Sci..

[2] Pandey H.O., Upadhyay D. (2022). Chapter Three—Global livestock production systems: Classification, status, and future trends. Emerging Issues in Climate Smart Livestock Production.

[3] Ortega D.L., Hong S.J., Wang H.H., Wu L. (2016). Emerging markets for imported beef in China: Results from a consumer choice experiment in Beijing. Meat Sci..

[4] Porto-Neto L.R., Sonstegard T.S., Liu G.E., Bickhart D.M., Da Silva M.V., Machado M.A., Utsunomiya Y.T., Garcia J.F., Gondro C., Van Tassell C.P. (2013). Genomic divergence of zebu and taurine cattle identified through high-density SNP genotyping. BMC Genom..

[5] Bickhart D.M., Xu L., Hutchison J.L., Cole J.B., Null D.J., Schroeder S.G., Song J., Garcia J.F., Sonstegard T.S., Van Tassell C.P. (2016). Diversity and population-genetic properties of copy number variations and multicopy genes in cattle. DNA Res..

[6] Scherf B.D., Pilling D. (2015). The Second Report on the State of the World’s Animal Genetic Resources for Food and Agriculture.

[7] FAO Shaping the future of livestock. Proceedings of the 10th Global Forum for Food and Agriculture (GFFA).

[8] Xu L., Yang L., Zhu B., Zhang W., Wang Z., Chen Y., Zhang L., Gao X., Gao H., Liu G.E. (2019). Genome-wide scan reveals genetic divergence and diverse adaptive selection in Chinese local cattle. BMC Genom..

[9] Wang M., Ding Y. (1996). The importance of work animals in rural China. World Anim. Rev..

[10] Chen Q., Zhan J., Shen J., Qu K., Hanif Q., Liu J., Zhang J., Chen N., Chen H., Huang B. (2020). Whole-genome resequencing reveals diversity, global and local ancestry proportions in Yunling cattle. J. Anim. Breed. Genet..

[11] Cozzi G., Brscic M., Gottardo F. (2009). Main critical factors affecting the welfare of beef cattle and veal calves raised under intensive rearing systems in Italy: A review. Ital. J. Anim. Sci..

[12] Nancy J.T., Nelson H.L. (2009). Effects of breed type and supplementation during grazing on carcass traits and meat quality of bulls fattened on improved savannah. Livest. Sci..

[13] Ji J., Yang H., Xu M.L. (2024). Amino Acid and Fatty Acid Profile of Hulunbuir Lambs under Different Grazing Intensities and Supplementary Feeding Levels. Acta Agrestia Sin..

[14] Chen B.Y., Liu Y., Chen R. (2021). Research on influence factors of inosine acid content in livestock and poultry muscle. J. Shaanxi Univ. Technol. Nat. Sci. Ed..

[15] Liu H.L., Zan L.S., Wang H.C. (2012). Chinese beef cattle improvement should pay attention to several technical links. Chin. J. Anim. Sci..

[16] Li S., Zou Y., Wang P., Qu M.R., Zheng W.B., Wang P., Chen X.Q., Zhu X.Q. (2021). Prevalence and multilocus genotyping of *Cryptosporidium* spp. in cattle in Jiangxi Province, southeastern China. Parasitol. Res..

[17] Li S., Wang P., Zhu X.Q., Zou Y., Chen X.Q. (2022). Prevalence and genotypes/subtypes of *Enterocytozoon bieneusi* and *Blastocystis* sp. in different breeds of cattle in Jiangxi Province, southeastern China. Infect. Genet. Evol..

[18] (2001). Hygienic Quarantine Rules for Slaughtering Livestock and Poultry.

[19] Serra X., Gil M., Gispert M., Guerrero L., Oliver M.A., Sañudo C., Campo M.M., Panea B., Olleta J.L., Quintanilla R. (2004). Characterisation of young bulls of the Bruna dels *Pirineus* cattle breed (selected from old Brown Swiss) in relation to carcass, meat quality and biochemical traits. Meat Sci..

[20] Carrillo J.A., Bai Y., He Y., Li Y., Cai W., Bickhart D.M., Liu G., Barao S.M., Sonstegard T., Song J. (2021). Growth curve, blood parameters and carcass traits of grass-fed Angus steers. Animal.

[21] Oattes J.L., Shao T., Henley P.A., Shike D.W. (2021). Fetal programming effects of early weaning on subsequent parity calf performance. Transl. Anim. Sci..

[22] Kiyanzad M.R. (2005). Comparison of carcass composition of Iranian fat-tailed sheep. Asian-Australas. J. Anim. Sci..

[23] Warren H.E., Scollan N.D., Enser M., Hughes S.I., Richardson R.I., Wood J.D. (2008). Effects of breed and a concentrate or grass silage diet on beef quality in cattle of 3 ages. I. Animal performance, carcass quality and muscle fatty acid composition. Meat Sci..

[24] Li S., Jin Y., Yan Z. (2016). Study on slaughtering performance and meat quality of yaks around lake. Food Ind. J..

[25] Swatland H.J. (1994). The cellular basis of postnatal muscle growth. Structure and Development of Meat Animals and Poultry.

[26] Xie X.X., Meng Q.X., Ren L.P., Shi F.H., Zhou B. (2012). Effect of cattle breed on finishing performance, carcass characteristics and economic benefits under typical beef production system in China. Ital. J. Anim. Sci..

[27] Ngapo T.M., Dransfield E. (2006). British consumers preferred fatness levels in beef: Surveys from 1955, 1982 and 2002. Food Qual. Prefer..

[28] Tran D., Thu N. (2006). Meat quality: Understanding of meat tenderness and influence of fat content on meat flavor. J. Sci. Technol. Dev..

[29] Uğurlu M., Ekiz B., Teke B. (2017). Meat quality traits of male Herik lambs raised under an intensive fattening system. Turk. J. Vet. Anim. Sci..

[30] Carpenter C.E., Cornforth D.P., Whittier D. (2001). Consumer preferences for beef color and packaging did not affect eating satisfaction. Meat Sci..

[31] Joo S.T., Kim G.D., Hwang Y.H., Ryu Y.C. (2013). Control of fresh meat quality through manipulation of muscle fiber characteristics. Meat Sci..

[32] Renerre M. (1982). Factors involved in the discoloration of beef meat. Int. J. Food Sci. Technol..

[33] Jeong J.Y., Hur S.J., Yang H.S., Moon S.H., Hwang Y.H., Park G.B., Joo S.T. (2009). Discoloration characteristics of 3 major muscles from cattle during cold storage. J. Food Sci..

[34] Boles J.A., Pegg R. (2010). Meat Color, Montana State University and Saskatchewan Food Product Innovation, Program University of Saskatchewan. https://docobook.com/meat-color-safe-spectrum.html.

[35] Toldrá F. (2006). Meat: Chemistry and biochemistry. Handbook of Food Science, Technology and Engineering.

[36] Vergara H., Molin A., Gallego L. (1999). Influence of sex and slaughter weight on carcass and meat quality in light and medium weight lambs produced in intensive systems. Meat Sci..

[37] Hultgren J., Segerkvist K.A., Berg C. (2022). Preslaughter stress and beef quality in relation to slaughter transport of cattle. Livest. Sci..

[38] Warriss P.D. (2010). Meat Science: An Introductory Text.

[39] Abebe G., Kannan G., Goetsch A.L. (2010). Effects of small ruminant species and origin (highland and lowland) and length of rest and feeding period on harvest measurements in Ethiopia. Afr. J. Agric. Res..

[40] Losada-Espinosa N., Estévez-Moreno L.X., Bautista-Fernández M. (2021). Cattle welfare assessment at the slaughterhouse level: Integrated risk profiles based on the animal’s origin, pre-slaughter logistics, and iceberg indicators. Prev. Vet. Med..

[41] Moore M.C., Gray G.D., Hale D.S. (2012). National Beef Quality Audit–2011: In-plant survey of targeted carcass characteristics related to quality, quantity, value, and marketing of fed steers and heifers. J. Anim. Sci..

[42] Geletu U.S., Usmael M.A., Mummed Y.Y. (2021). Quality of cattle meat and its compositional constituents. Vet. Med. Int..

[43] Marchello J.A., Dryden F.D. (1968). While Ideas differ Meat Quality Is defined. Progress. Agric. Ariz..

[44] Huffman K.L., Miller M.F., Hoover L.C., Wu C.K., Brittin H.C., Ramsey C.B. (1996). Effect of beef tenderness on consumer satisfaction with steaks consumed in the home and restaurant. J. Anim. Sci..

[45] Watanabe A., Daly C.C., Devine C.E. (1996). The effects of the ultimate pH of meat on tenderness changes during ageing. Meat Sci..

[46] Xu H.X., Luo W.J., Lei L. (2023). Nutritional composition analysis and quality evaluation of cattle in different regions of Guizhou Province (China). Czech J. Food Sci..

[47] Essen-Gustavsson B., Karlsson A., Lundström K. (1994). Intramuscular fat and muscle fibre lipid contents in halothane-gene-free pigs fed high or low protein diets and its relation to meat quality. Meat Sci..

[48] Tan Z., Jiang H. (2024). Molecular and Cellular Mechanisms of Intramuscular Fat Development and Growth in Cattle. Int. J. Mol. Sci..

[49] Mwangi F.W., Charmley E., Gardiner C.P. (2019). Diet and genetics influence beef cattle performance and meat quality characteristics. Foods.

[50] McMillin K.W., Hoffman L.C., Kerry J. (2009). Improving the quality of meat from ratites. Improving the Sensory and Nutritional Quality of Fresh Meat.

[51] Bouton P.E., Ford A.L., Harris P.V., Ratcliff D. (1975). Objective assessment of meat juiciness. J. Food Sci..

[52] Suleimenova A. (2016). Biochemical and Sensory Profile of Meat from Dairy and Beef Cattle. Master’s Thesis.

[53] Purslow P.P., Oiseth S., Hughes J. (2016). The structural basis of cooking loss in beef: Variations with temperature and ageing. Food Res. Int..

[54] Tornberg E.V.A. (2005). Effects of heat on meat proteins–Implications on structure and quality of meat products. Meat Sci..

[55] Choi Y.M., Kim B.C. (2009). Muscle fiber characteristics, myofibrillar protein isoforms, and meat quality. Livest. Sci..

[56] Reichhardt C.C., Feuz R., Brady T.J., Motsinger L.A., Briggs R.K., Bowman B.R., Garcia M.D., Larsen R., Thornton K.J. (2021). Interactions between cattle breed type and anabolic implant strategy impact circulating serum metabolites, feedlot performance, feeding behavior, carcass characteristics, and economic return in beef steers. Domest. Anim. Endocrin.

[57] De Souza R.J., Mente A., Maroleanu A. (2015). Intake of saturated and trans unsaturated fatty acids and risk of all cause mortality, cardiovascular disease, and type 2 diabetes: Systematic review and meta-analysis of observational studies. BMJ.

[58] Wood J.D., Richardson R.I., Nute G.R. (2004). Effects of fatty acids on meat quality: A review. Meat Sci..

[59] Davis H., Magistrali A., Butler G. (2022). Nutritional benefits from fatty acids in organic and grass-fed beef. Foods.

[60] World Health Organization (2003). Diet, Nutrition and the Prevention of Chronic Diseases.

[61] Scollan N., Hocquette J.F., Nuernberg K. (2006). Innovations in beef production systems that enhance the nutritional and health value of beef lipids and their relationship with meat quality. Meat Sci..

[62] Dinh T.T., Blanton J.R., Riley D.G., Chase C.C., Coleman S.W., Phillips W.A., Brooks J.C., Miller M.F., Thompson L.D. (2010). Intramuscular fat and fatty acid composition of longissimus muscle from divergent pure breeds of cattle. J. Anim. Sci..

[63] Khan M.I., Jo C., Tariq M.R. (2015). Meat flavor precursors and factors influencing flavor precursors—A systematic review. Meat Sci..

[64] Sturdivant C.A., Lunt D.K., Smith G.C. (1992). Fatty acid composition of subcutaneous and intramuscular adipose tissues and M. longissimus dorsi of Wagyu cattle. Meat Sci..

[65] Joint WHO/FAO/UNU Expert Consultation (2007). Protein and Amino Acid Requirements in Human Nutrition: Report of a Joint FAO/WHO/UNU Expert Consultation.

[66] Holló G., Csapó J., Szűcs E., Tőzsér J., Repa I., Holló I. (2001). Influence of Breed, Slaughter Weight and Gender on Chemical Composition of Beef. Part 1. Amino Acid Profile and Biological Value of Proteins. Asian-Australas. J. Anim. Sci..

[67] Vopálenský Josef Suchý P., Straková E., Šimek F., Macháček M., Herzig I. (2017). Amino acid levels in muscle tissue of eight meat cattle breeds. Czech J. Anim. Sci..

[68] Calkins C.R., Hodgen J.M. (2007). A fresh look at meat flavor. Meat Sci..

[69] Rustan A.C., Drevon C.A. (2005). Fatty acids: Structures and properties. eLS.

[70] Corbin C.H., O’Quinn T.G., Garmyn A.J., Legako J.F., Hunt M.R., Dinh T.T.N., Rathmann R.J., Brooks J.C., Miller M.F. (2015). Sensory evaluation of tender beef strip loin steaks of varying marbling levels and quality treatments. Meat Sci..

[71] Melton S.L., Black J.M., Davis G.W. (1982). Flavor and selected chemical components of ground beef from steers backgrounded on pasture and fed corn up to 140 days. J. Food Sci..

[72] Daley C., Abbott A., Doyle P., Nader G., Larson S. (2010). A review of fatty acid profiles and antioxidant content in grass-fed and grain-fed beef. Nutr. J..

[73] Ma D., Kim Y.H.B., Cooper B. (2017). Metabolomics profiling to determine the effect of postmortem aging on color and lipid oxidative stabilities of different bovine muscles. J. Agric. Food Chem..

[74] Nyquist K.M., O’Quinn T.G., Drey L.N., Lucherk L.W., Brooks J.C., Miller M.F., Legako J.F. (2018). Palatability of beef chuck, loin, and round muscles from three USDA quality grades. J. Anim. Sci..

[75] Yancey E.J., Grobbel J.P., Dikeman M.E. (2006). Effects of total iron, myoglobin, hemoglobin, and lipid oxidation of uncooked muscles on livery flavor development and volatiles of cooked beef steaks. Meat Sci..

[76] Miller R.K., Luckemeyer T.J., Kerth C.R. (2023). Descriptive beef flavor and texture attributes relationships with consumer acceptance of US light beef eaters. Meat Sci..

